# *GUCA1A* mutation causes maculopathy in a five-generation family with a wide spectrum of severity

**DOI:** 10.1038/gim.2016.217

**Published:** 2017-01-26

**Authors:** Xue Chen, Xunlun Sheng, Wenjuan Zhuang, Xiantao Sun, Guohua Liu, Xun Shi, Guofu Huang, Yan Mei, Yingjie Li, Xinyuan Pan, Yani Liu, Zili Li, Qingshun Zhao, Biao Yan, Chen Zhao

**Affiliations:** 1 Department of Ophthalmology and Vision Science, Eye & ENT Hospital, Shanghai Medical College, Fudan University, Shanghai, China; 2 Department of Ophthalmology, The First Affiliated Hospital of Nanjing Medical University, State Key Laboratory of Reproductive Medicine, Nanjing, China; 3 Key Laboratory of Myopia of State Health Ministry (Fudan University) and Shanghai Key Laboratory of Visual Impairment and Restoration, Shanghai, China; 4 Department of Ophthalmology, Ningxia Eye Hospital, People Hospital of Ningxia Hui Autonomous Region (First Affiliated Hospital of Northwest University for Nationalities), Yinchuan, China; 5 Department of Ophthalmology, Children’s Hospital of Zhengzhou, Zhengzhou, China; 6 Department of Ophthalmology, Qilu Hospital of Shandong University, Jinan, China; 7 Department of Ophthalmology, The Third Affiliated Hospital of Nanchang University, Nanchang, China; 8 MOE Key Laboratory of Model Animal for Disease Study, Model Animal Research Center, Nanjing University, Nanjing, China; 9 Research Center, Eye & ENT Hospital, Shanghai Medical College, Fudan University, Shanghai, China; 10 State Key Laboratory of Ophthalmology, Zhongshan Ophthalmic Center, Sun Yat Sen University, Guangzhou, China

**Keywords:** *GUCA1A*, maculopathy, medical genetics, ophthalmology, pathogenic mechanism

## Abstract

**Purpose:**

The aim of this study was to investigate the genetic basis and pathogenic mechanism of variable maculopathies, ranging from mild photoreceptor degeneration to central areolar choroidal dystrophy, in a five-generation family.

**Methods:**

Clinical characterizations, whole-exome sequencing, and genome-wide linkage analysis were carried out on the family. Zebrafish models were used to investigate the pathogenesis of *GUCA1A* mutations.

**Results:**

A novel mutation, *GUCA1A* p.R120L, was identified in the family and predicted to alter the tertiary structure of guanylyl cyclase-activating protein 1, a photoreceptor-expressed protein encoded by the *GUCA1A* gene. The mutation was shown in zebrafish to cause significant disruptions in photoreceptors and retinal pigment epithelium, together with atrophies of retinal vessels and choriocapillaris. Those phenotypes could not be fully rescued by exogenous wild-type *GUCA1A*, suggesting a likely gain-of-function mechanism for p.R120L. *GUCA1A* p.D100E, another mutation previously implicated in cone dystrophy, also impaired the retinal pigment epithelium and photoreceptors in zebrafish, but probably via a dominant negative effect.

**Conclusion:**

We conclude that *GUCA1A* mutations could cause significant variability in maculopathies, including central areolar choroidal dystrophy, which represents a severe pattern of maculopathy. The diverse pathogenic modes of *GUCA1A* mutations may explain the phenotypic diversities.

*Genet Med* advance online publication 26 January 2017

## Introduction

Inherited retinal degenerations (IRDs) include a group of diverse retinal degenerative diseases presenting both genetic and clinical heterogeneities. To date, according to RetNet (https://sph.uth.edu/retnet/), 293 loci (including 256 identified genes) have been associated with IRDs. Among the 256 identified genes, the guanylate cyclase activator 1A gene (*GUCA1A*; MIM 600364) has been implicated in dominant cone dystrophy, cone-rod dystrophy, and macular dystrophy.^1,2,3^ *GUCA1A*, located in 6p21.1, encodes the guanylyl cyclase-activating protein 1 (GCAP1), a photoreceptor-specific protein with more expression in the inner segment/outer segment (OS) layer of cones than rods in mammals.^4^ GCAP1 is a critical component in the phototransduction cascade, which acts as a calcium sensor in the recovery of photoreceptors from photon capture by regulating the retinal guanylate cyclase 1 (retGC1) –medicated cyclic guanosine monophosphate production in a calcium-sensing manner.^5,6^ RetGC1 is encoded by the guanylate cyclase 2D gene (*GUCY2D*; MIM 600179). The important roles of GCAP1 and retGC1 in regulating hemostasis of calcium and cyclic guanosine monophosphate in photoreceptors have been well addressed.

In this study we found a novel *GUCA1A* mutation to be disease causative in a five-generation family affected with variable maculopathies ranging from mild photoreceptor degeneration to central areolar choroidal dystrophy (CACD). CACD is a special form of IRD that mainly affects the maculae and is characterized by a well-defined atrophic region of retinal pigment epithelium (RPE) and choriocapillaris at the latest stage.^7,8,9^ Before our study, mutations in two other genes, peripherin-2 (*PRPH2*; MIM 179605) and *GUCY2D*, were implicated in CACD etiology.^8,10,11,12^ We also found that diverse pathogenic mechanisms of *GUCA1A* mutations might correlate with the phenotypic diversity of IRDs.

## Materials and Methods

### Participants and clinical assessments

**Figure 1:**
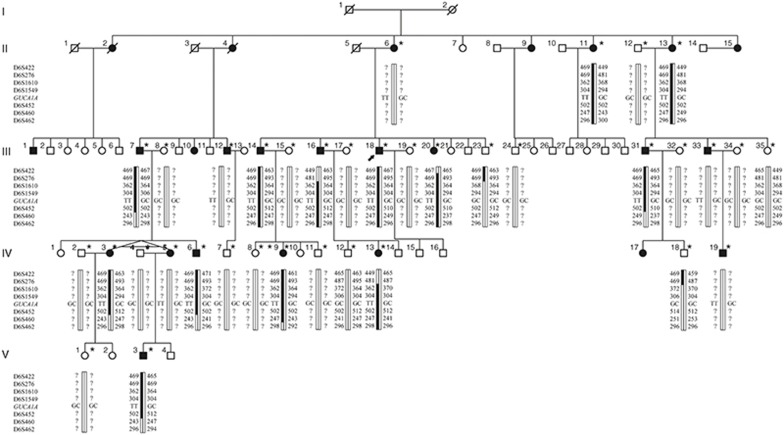
**Pedigree of family DC and haplotype reconstruction for the mapped region on chromosome 6 in family DC.** Filled and open symbols represent affected and unaffected members, respectively. The proband is indicated by the black arrow. Haplotypes for tested short tandem repeat (STR) markers in the mapped region and those flanking it, and genotypes for *GUCA1A* c.359_360, are given for all participants. Black bars represent the ancestral haplotype associated with the disease. *Individuals from whom blood samples were collected. The mapped region flanked by markers D6S276 and D6S460 was shared by all patients and was absent in all unaffected members.

Our study conformed to the Declaration of Helsinki and was prospectively reviewed and approved by the ethics committee of Ningxia Eye Hospital. Written informed consent was obtained from all participants before their enrollment. Eighteen patients and 18 unaffected family members from family DC were included in the study (**Figure 1**). Medical records from each participant were reviewed. Routine ophthalmic examination was conducted for all participants, and all patients received comprehensive ophthalmic examinations, including best-corrected visual acuity, slit-lamp examination, visual field test, funduscopic evaluations, and electroretinograms. Fundus autofluorescence, fundus fluorescein angiography, optical coherence tomography, and electroretinography were performed when possible. Another 423 unrelated Chinese controls free of maculopathy and other major ocular diseases were also recruited. Samples of peripheral venous blood (5 ml) were collected from each participant for genomic DNA extraction using a QIAmp DNA Mini Blood Kit (Qiagen, Hilden, Germany).

### Linkage analysis

Thirteen patients from family DC (II:11, II:13, III:7, III:14, III:16, III:18, III:20, III:31, IV:3, IV:6, IV:9, IV:13, and V:3) and four unaffected members from the same family (III:23, III:35, IV:12, and IV:18) were carefully examined and genotyped. Genome-wide linkage analysis was conducted in collaboration with Genesky Biotech (Shanghai, China) using a total of 366 microsatellite markers spaced at about 10 cM (Weber set 6.0) and distributed throughout all autosomes; these markers were amplified by polymerase chain reaction (PCR) using primers labeled by FAM.^13,14^ PCR products were appropriately pooled according to allele size and labeling, mixed with GeneScan-500 TAMRA standard (Applied Biosystems, Foster City, CA), denatured, loaded onto 6% standard denaturing polyacrylamide gels, and run in an ABI 3130xl sequencer (Applied Biosystems) for fluorescent detection. Genotype calling was performed using the GeneMapper 4.1 software package (Applied Biosystems). The pedigree showed male-to-male transmission of the disease and an approximately 1:1 ratio between affected males and females, indicating an autosomal-dominant mode of inheritance. Therefore, the multipoint log odds score was calculated using an autosomal-dominant inheritance mode with a risk allele frequency of 0.0001 and a penetrance of 99% (LINKAGE software package of MERLIN 1.1.2). Family and haplotype data were generated using Cyrillic software (version 2.1) and confirmed by inspection.

### Exome sequencing and bioinformatics analysis

Two patients, DC-IV:3 and DC-IV:13, were selected for whole-exome sequencing using the SureSelect Human All Exon 50Mb Kit (Agilent Technologies, Santa Clara, CA) and the HiSeq 2000 platform (Illumina, San Diego, CA).^15^ Illumina base-calling software v1.7 was then applied to turn raw image files into 90-base-paired-end reads. Bioinformatics were subsequently analyzed, and mutations validated.^16,17^ Briefly, all detected variants were initially filtered against six single-nucleotide polymorphism databases, including dbSNP144 (http://hgdownload.cse.ucsc.edu/goldenPath/hg19/database/snp144.txt.gz), the HapMap project (ftp://ftp.ncbi.nlm.nih.gov/hapmap), the 1000 Genomes Project (ftp://ftp.1000genomes.ebi.ac.uk/vol1/ftp), the YanHuang database (http://yh.genomics.org.cn/), the Exome Variant Server (http://evs.gs.washington.edu/EVS/), and the Exome Aggregation Consortium database (http://exac.broadinstitute.org/). Noncoding variants were discarded, and only variants located within an annotated exon or within 10 base pairs on either side were analyzed further. Intrafamilial cosegregation analysis and prevalence testing in the 423 unrelated controls were then conducted with the primers detailed in **Supplementary Table S1** online.

### In silico analyses

Evolutionary conservation of the mutated residues was analyzed with Vector NTI Advance 2011 (Invitrogen, Carlsbad, CA) by aligning the protein sequence of human GCAP1 (NP_000400.2) with sequences of the following orthologous proteins: *Pan troglodytes* (ENSPTRP00000031039), *Canis lupus familiaris* (ENSCAFP00000002385), *Bos taurus* (NP_776971.1), *Sus scrofa* (ENSSSCP00000001774), *Mus musculus* (NP_032215.2), *Gallus gallus* (NP_989651), and *Danio rerio* (NP_571945.1). Crystal structural models of the wild-type (WT) and mutant GCAP1 were constructed using the SWISS-MODEL online server. Predicted structures were displayed by PyMol software (version 1.5).

### Plasmids construction and messenger RNA synthesis

The open reading frame sequence of WT human *GUCA1A* (NM_000409.3) was synthesized and inserted into the pXT7 plasmids (a gift from Anming Meng, Tsinghua University) to get the linearized AcpXT7-GUCA1A^WT^ plasmid for in vivo transcription.^18^ Missense *GUCA1A* mutations, p.R120L and p.D100E, were introduced into the generated WT plasmid using a QuikChange Lightning Site-Directed Mutagenesis Kit (Agilent Technologies) to obtain recombinant plasmids AcpxT7-GUCA1A^R120L^ and AcpxT7-GUCA1A^D100E^. The inserted sequences of all produced plasmids were validated via Sanger sequencing in both directions. Capped and tailed messenger RNAs (mRNAs) of human *GUCA1A*^WT^, *GUCA1A*^p.R120L^, and *GUCA1A*^p.D100E^ were then generated using a mMESSAGE mMACHINE T7 Ultra Kit (Ambion, Austin, TX), and purified with a RNeasy Kit (Qiagen).^18^

### Zebrafish manipulations

Zebrafish (Tubingen strain) experiments were performed in accordance with the Institutional Animal Care and Use Committee–approved protocol in the Model Animal Research Center, Nanjing University, China. One- to 2-cell zebrafish embryos (0 days postfertilization (dpf)) were randomly divided into three groups and microinjected with 1 nl of solution containing 150 pg purified designate mRNA. Embryos with systemic deformities were discarded. Only embryos with a normal systemic appearance were included for further investigations. The investigator was blinded to the group allocation during the experiment and when assessing the outcome.

### Reverse transcriptase PCR and real-time PCR

Total RNA was isolated using Trizol (Invitrogen), followed by reverse transcription PCR for complementary DNA synthesis with a reverse transcription kit (Invitrogen). Real-time PCR was conducted using FastStart Universal SYBR Green Master (ROX; Roche, Basel, Switzerland) with the StepOne Plus Real-time PCR System (Applied Biosystems). Primer information is listed in **Supplementary Table S1** online.

### Transmission electron microscopy and immunofluorescent staining

Morphological changes of the zebrafish eyes from the different injected groups at 4 dpf were visualized with a Leica DM-IL microscope (Leica, Wetzlar, Germany). Transmission electron microscopy (JEOL, Tokyo, Japan) was applied for ultrastructural analysis of the eye in larvae 11 dpf.^19^ Retinal sections of zebrafish at 4 dpf were obtained for immunofluorescent staining to visualize RPE, rod, and cone photoreceptors.^18^ RPE was immunostained with the RPE-specific antibody Zpr-2. As for the immunofluorescent staining of photoreceptors, we labeled cone photoreceptor OS//inner segment with peanut agglutinin lectin, and cone cell body with an antibody against Zpr-1, a marker for red/green cones.^15^ Rod photoreceptors were visualized by staining of rhodopsin. Information about antibodies is detailed in **Supplementary Table S2** online. The terminal deoxyribonucleotidyl transferase–mediated 2′-deoxyuridine 5′-triphosphate–digoxigenin nick end labeling (TUNEL) assay was applied for detection of DNA fragmentation and investigation of active cell death (apoptosis). We used a transgenic zebrafish (*flk1*: enhanced green fluorescent protein (EGFP)) to visualize the systemic vessels, including ocular vessels.^20,21^ Truncal vasculature of living zebrafish was visualized with inverted fluorescent microscopy (Leica DM IL), while the ocular vessels in living zebrafish were visualized using a Leica TCS SP5 confocal system.

### Statistics

GraphPad Prism (version 4.0; GraphPad Software, San Diego, CA) was used for statistical analysis. We applied one-way analysis of variance or the Student’s *t*-test for comparisons between different groups. Data were presented as mean ± SEM, and *P* < 0.05 was considered statistically significant.

## Results

### Clinical presentations

**Figure 2:**
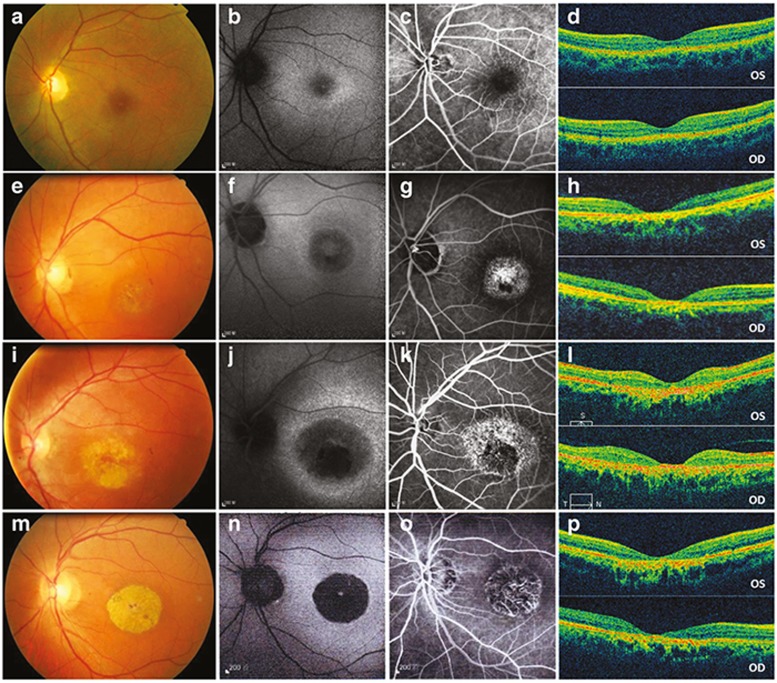
**Fundus photography, fundus autofluorescence (FAF), fundus fluorescein angiography (FFA), and optical coherence tomography (OCT) presentations for patients with different grades of maculopathy.** (**a–d**) Patient DC-IV:6 with grade I maculopathy showed slight parafoveal hypopigmentation (**a**) with discrete increased FAF (**b**) and hyperfluorescent parafoveal changes on FFA (**c**). OCT revealed that the outer nuclear layer (ONL) and inner segment/outer segment (IS/OS) layer are slightly reduced in the fovea. The retinal pigment epithelium (RPE) layer was relatively normal (**d**). (**e–h**) Patient DC-III:16 with grade II maculopathy showed pigmental disturbance in the fundus (**e**) and speckled changes of increased fluorescence in the maculae, with a round area of hypofluorescence seen on FAF and FFA (**f**, **g**). OCT showed that the ONL and IS/OS layer were almost vanished in the fovea and its surrounding maculae, with no remarkable changes detected in the peripheral retina. Macular RPE was slightly changed (**h**). (**i–l**) In patient DC-III:14 with grade III maculopathy, a patch of circumscribed chorioretinal atrophy outside the central fovea is indicated on a fundus photograph. Slight hypopigmentation is found within this lesion (**i**). This area shows severely decreased to absent FAF (**j**), with choroidal vessels visible on FFA (**k**). OCT demonstrated that the ONL and IS/OS layer in the fovea had vanished. Macular RPE was moderately changed (**l**). (**m–p**) In patient DC-III:18 with grade IV maculopathy, the fundus photograph presents a well-demarcated area of chorioretinal atrophy involving the fovea (**m**), corresponding to an area of absent FAF (**n**). FFA shows a well-defined area of chorioretinal atrophy with a few large choroidal vessels visible (**o**). OCT shows a vanished ONL and IS/OS layer in the fovea. Macular RPE degeneration was the severest of all (**p**). OD = right eye; OS = left eye.

Family DC is a large, five-generation Chinese family recruited from Ningxia Eye Hospital. A total of 18 patients with maculopathy and 18 unaffected family members participated in our study (**Figure 1**). All patients received detailed ophthalmic examinations; the results are summarized in **Supplementary Table S3** online. All affected eyes of the 18 patients were graded as I to IV based on the severity of maculopathy as revealed by their fundus presentations. Briefly, grade I is characterized by subtle photoreceptor degeneration. In grade II, moderate photoreceptor degeneration with RPE attenuation is observed. Grade III is marked by one or more patches of RPE and choriocapillary atrophy, making choroidal vessels visible. Significant atrophy of the outer nuclear layer, RPE, and choriocapillaries in a well-defined region surrounding the fovea is characteristic of grade IV, leading to a remarkable decrease in visual acuity. Among the 36 eyes analyzed, 8 were defined as grade I, 6 as grade II, 15 as grade III, and 7 as grade IV. The eyes with grade III and IV maculopathy present the typical features of CACD, which represents a unique and severe pattern of maculopathy. Thus, those eyes are clinically diagnosed as CACD. Fundus photography, fundus autofluorescence, fundus fluorescein angiography, optical coherence tomography, and electroretinography for patients with different grades of maculopathy are summarized in **Figure 2**, **Supplementary Figure S1** online, and **Supplementary Table S4** online.

Sixteen of the 18 included patients experienced color anomaly, whereas no patient showed photophobia or nyctalopia. The initial symptom for most patients in this family was vision loss, whereas for patients DC-IV:9 and DC-V:3, their disease status was revealed upon ophthalmic examination. Most patients in this family claimed vision loss started in their 20s or 30s. DC-IV:19 reported poor vision since early childhood. It was thus difficult to determine his exact age at onset of vision loss. Age at onset of vision loss in patient DC-III:14 was 40 years old, which was the latest within this family. Disease progression for patients in this family varied. Patients DC-IV:3 and DC-IV:5 progressed rapidly after their initial symptom, while the disease course for patients DC-III:20 and DC-III:14 was relatively slow.

### Linkage analysis

To determine the genetic lesion of maculopathy in this family, we carried out a genome-wide linkage analysis on 13 patients and 4 unaffected members by using 366 microsatellite markers distributed throughout the whole genome (**Figure 1**). Linkage analysis revealed a candidate region cosegregating with the disease on chromosome 6. The critical interval was flanked by markers D6S276 and D6S460, with a maximum log odds score of 4.54 at D6S1610 (**Supplementary Table S5** online).

### Exome sequencing

**Figure 3:**
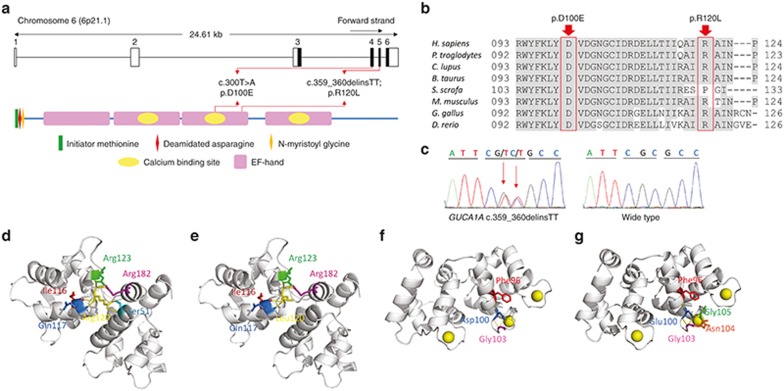
***GUCA1A* mutations investigated in this study.** (**a**) Schematic representations of the relative linear locations of *GUCA1A* p.D100E and p.R120L mutations in the context of the genome structure (top) and protein structure (bottom) are shown. Domains of the guanylyl cyclase-activating protein 1 (GCAP1) are also annotated. (**b**) Orthologous protein sequence alignment of GCAP1 from multiple species. Conserved residues are shaded. Mutated residues D100 and R120 are boxed. (**c**) Sequencing chromatogram of the identified *GUCA1A* c.359_360delinsTT mutation (top). Wild-type sequences are shown at the bottom. (**d,e**) Crystal structures of wild-type (**d**) and mutant human GCAP1 carrying p.R120L (**e**). The mutational spot and its generated hydrogen bonds are highlighted yellow. Amino acids interacted with residue 120, including Ser51, Ile116, Gln117, Arg123, and Arg182, are indicated. The hydrogen bond between residue 120 and Ser51 is eliminated upon the change from wild-type arginine to mutant leucine. (**f,g**) Crystal structures of wild-type (**f**) and mutant GCAP1 carrying p.D100E (**g**). The mutated residue is indicated in blue. Its generated hydrogen bonds and interacted proteins are annotated. Two hydrogen bonds between residue 100 and Gly105/Asn104 are generated as a result of the mutation.

Whole-exome sequencing was subsequently performed on two patients, DC-IV:3 and DC-IV:13, to determine the disease-causative mutation in this family. The mean depths of targeted regions were 81-fold and 103.8-fold for patients DC-IV:3 and DC-IV:13, respectively. A total of 127,904 variants were detected by whole-exome sequencing, and these were subjected to bioinformatics analysis, filtering, and validation by Sanger sequencing using the primers detailed in **Supplementary Table S1** online. Among all variants, only one heterozygous variant, c.359_360delinsTT in the *GUCA1A* gene (MIM 600364), was deemed to be likely pathogenic (**Figure 3c**). The variant c.359_360delinsTT cosegregated with the disease phenotype in the family (**Figure 1**) and was absent in 423 additional ethnically matched, unrelated controls and five single-nucleotide polymorphism databases. This variant was located in exon 3 of the *GUCA1A* gene and is predicted to result in an amino acid substitution from arginine to leucine at residue 120 (p.R120L) of GCAP1, the protein encoded by the *GUCA1A* gene (**Figure 3a**). More important, our whole-exome sequencing results were consistent with the linkage data because the *GUCA1A* gene was located within the critical interval on chromosome 6 (**Figure 1**).

### Pathogenic analysis

The mutation *GUCA1A* p.R120L was conserved among all species aligned except for *S. scrofa* (**Figure 3b**), and was located in third EF-hand domain, which is involved in calcium binding (**Figure 3a**). To predict the pathogenic effect caused by this mutation, we performed crystal structural modeling for the mutant GCAP1 using SWISS-MODEL based on a preexisting structure of WT GCAP1 (Protein Data Bank identifier: 2R2I). Using this assay, we found that a hydrogen bond between residues Ser51 and Arg120 in the WT protein was eliminated in the mutated protein as a result of the replacement of arginine by leucine (**Figure 3d**,**e**). This hydrogen bond seems to be important for the tertiary structure of the protein because it connects α-helices of the second and the third EF-hand domains. Thus, it is very likely that the mutation p.R120L affects the folding and relevant biological properties of GCAP1.

To better determine the pathogenesis of *GUCA1A* p.R120L and to explain the phenotypic diversity caused by *GUCA1A* mutations, we introduced another previously reported mutation, p.D100E, which was reported to be correlated with cone dystrophy.^22,23^ This mutation, situated in the calcium-binding loop of the third EF hand, was conserved among all tested species (**Figure 3a**,**b**). A possible molecular structure of this mutant protein was also constructed based on the GCAP1 template, which indicated that this mutation would generate two novel hydrogen bonds between residue 100 and Asn104 and Gly105, respectively (**Figure 3f**,**g**).

### *GUCA1A* p.R120L causes photoreceptor impairments

We first measured whether *GUCA1A* p.R120L could cause photoreceptor degeneration. Previous studies have revealed the dominant nature of *GUCA1A* mutations.^24^ Consistent with this, no remarkable change was found in the eyes of mice homozygous for a null allele of *Guca1a*.^25^ To test the hypothesis that p.R120L causes disease through a gain-of-function mechanism, we overexpressed WT (hereafter termed *GUCA1A*^WT^) or mutated human *GUCA1A* mRNA (*GUCA1A*^p.R120L^) in zebrafish to characterize their relevant pathology. We also injected both *GUCA1A*^WT^ and *GUCA1A*^p.R120L^ into a group of zebrafish (*GUCA1A*^WT+p.R120L^) to assess whether the pathogenic impact of *GUCA1A*^p.R120L^ could be partly or fully rescued by WT mRNA. In this way, we sought to determine whether *GUCA1A*^p.R120L^ functions in a toxic gain-of-function or a dominant negative manner.

**Figure 4:**
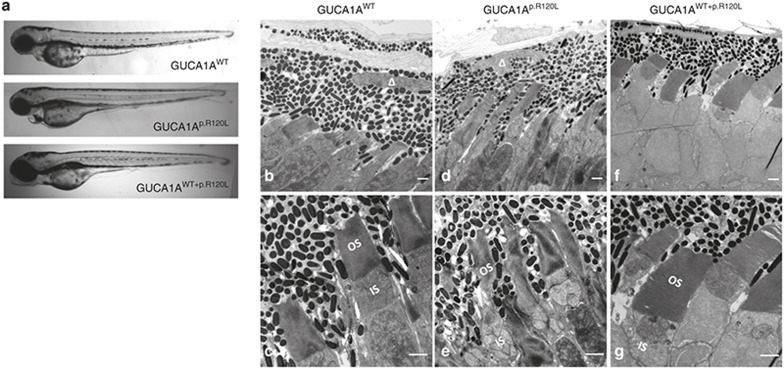
**Morphological changes caused by *GUCA1A* p.R120L.** (**a**) Light microscopy indicates no evident morphological changes in eyes of zebrafish injected with *GUCA1A*^WT^, or *GUCA1A*^p.R120L^, or *GUCA1A*^WT+p.R120L^ 4 days postfertilization (dpf). (**b–e**) Transmission electron microscopy of the retina of zebrafish 11 dpf injected with *GUCA1A*^WT^ or *GUCA1A*^p.R120L^. Photoreceptors are regularly lined and shaped in the *GUCA1A*^WT^-injected group (**b**, **d**). Thickness of the RPE; shrinking, twisty, and caducous photoreceptor outer segments; and choriocapillary disruptions are found in the *GUCA1A*^p.R120L^-injected group (**c**, **e**). ▵ represents the RPE nucleus. Scale bar = 1 μm.

Overexpression of exogenous proteins often causes systemic developmental defects as a result of toxicity; therefore, we only included zebrafish without systemic deformities for further analysis. Light microscopy revealed no evident morphological changes in the eyes of zebrafish injected with either *GUCA1A*^WT^, *GUCA1A*^p.R120L^, or *GUCA1A*^p.D100E^ at 4 dpf (**Figure 4a**). We conducted ultrastructural analysis of zebrafish at 11 dpf using transmission electron microscopy. According to transmission electron microscopy, the OSs and inner segments of photoreceptors in the *GUCA1A*^p.R120L^-injected zebrafish were irregular, with a shrinking and twisty appearance (**Figure 4d**,**e**).

**Figure 5:**
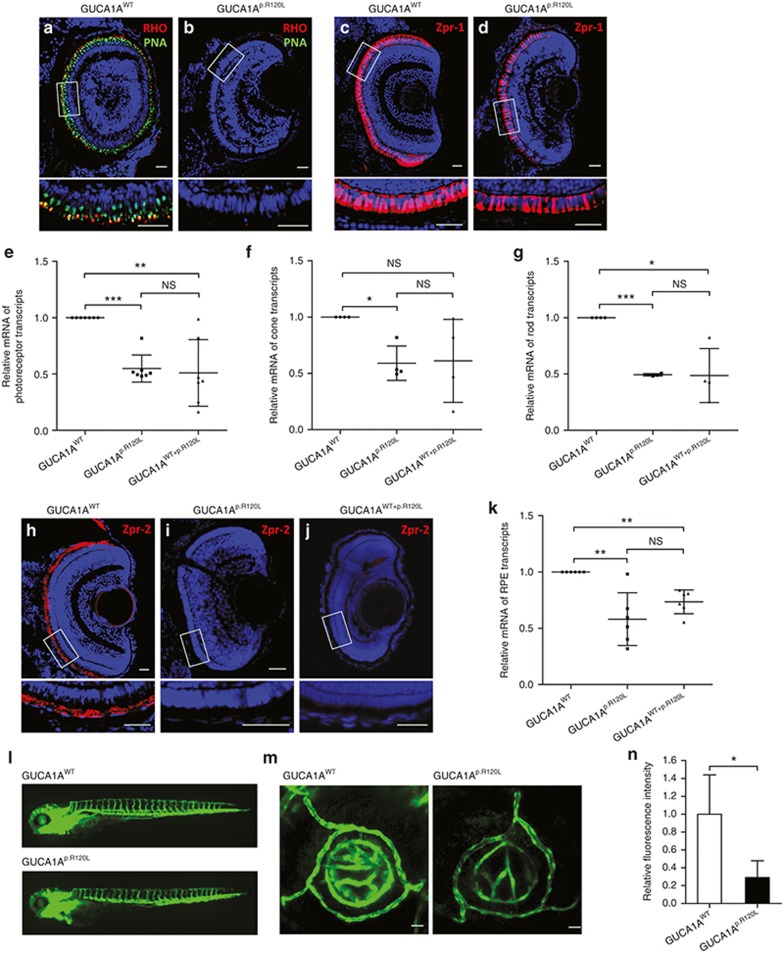
**Impairments in photoreceptors, the retinal pigment epithelium (RPE), and ocular vasculature induced by *GUCA1A* p.R120L in zebrafish.** (**a–d**) Immunofluorescent staining of rhodopsin (RHO) (red; **a,b**), peanut agglutinin (PNA) lectin (green; **a,b**), and Zpr-1 (red; **c,d**). RHO and PNA expressions were significantly reduced in zebrafish overexpressing *GUCA1A*^p.R120L^ (**a,b**). Zpr-1 expression was only slightly reduced in the *GUCA1A*^p.R120L^-injected group (**d**) when compared with *GUCA1A*^WT^-injected zebrafish (**c**). (**e–g**) Relative messenger RNA (mRNA) levels of photoreceptor-, cone-, and rod-specific transcripts in *GUCA1A*^p.R120L^- and *GUCA1A*^WT+p.R120L^-injected zebrafish compared with the *GUCA1A*^WT^-injected group. (**h–j**) Robust Zpr-2 expression was detected in retinal frozen sections of 4-dpf larvae injected with *GUCA1A*^WT^ (**h**), whereas Zpr-2 expression was diminished in the *GUCA1A*^p.R120L^-injected (**i**) and *GUCA1A*^WT+p.R120L^-injected (**j**) groups. (**k**) Relative mRNA levels of RPE characteristic transcripts in the *GUCA1A*^p.R120L^- and *GUCA1A*^WT+p.R120L^-injected groups were decreased when compared with the *GUCA1A*^WT^-injected zebrafish. (**l**) No truncal vascular anomaly is revealed in transgenic (*flk1*: enhanced green fluorescent protein (EGFP)) zebrafish injected with *GUCA1A*^p.R120L^. (**m**) Axial projections of confocal images from the two injected groups. Fluorescent intensity of EGFP is significantly reduced in the *GUCA1A*^p.R120L^-injected group. (**n**) The fluorescent intensity of EGFP calculated by ImageJ in the two groups. Results were obtained from technical triplicates. Error bars represent the SE. Error bars represent the SD. NS, not significant. *P < 0.05; **P < 0.01; ***P < 0.001. Scale bar = 20 mm.

Retinal sections of zebrafish larvae at 4 dpf were obtained for immunofluorescent staining. Consistent with ultrastructural changes, the cone inner segment/OS, indicated by peanut agglutinin lectin staining, were significantly reduced or even vanished in larvae injected with *GUCA1A*^p.R120L^ (**Figure 5a**,**b**). The red/green cone cell bodies were also affected by the mutation: a reduction of 73% of fluorescent intensity of Zpr-1 staining was revealed in the *GUCA1A*^p.R120L^-injected larvae compared with the *GUCA1A*^WT^-injected group (**Figure 5c**,**d**). Rhodopsin staining was nearly undetectable in fish injected with *GUCA1A*^p.R120L^, suggesting that the mutation causes similar defects in rods (**Figure 5a**,**b**). In addition, mRNA levels of photoreceptor-specific genes were also found to be decreased in *GUCA1A*^p.R120L^-injected embryos and could not be rescued (**Figure 5e**). Subgroup analysis further suggested that both cones (**Figure 5f**) and rods (**Figure 5g**) were affected. Thus, our findings revealed that photoreceptors are impaired by *GUCA1A* p.R120L in a toxic gain-of-function way.

### *GUCA1A* p.R120L induces RPE degeneration in zebrafish

We next measured whether RPE cells were affected by this mutation. When compared with the *GUCA1A*^WT^-injected group, we found RPE cells in both *GUCA1A*^p.R120L^- and *GUCA1A*^WT+p.R120L^-injected zebrafish were remarkably thinned with altered nuclei, suggesting atrophic changes (**Figure 4b**–**g**). Retinal sections of zebrafish larvae at 4 dpf were obtained for immunofluorescent staining. Despite dramatic morphological changes, we did not find any apoptotic cells in the RPE layer as determined by TUNEL staining (data not shown). However, we found that the reactivity of Zpr-2, an RPE characteristic antibody, was diminished in the RPE of larvae expressing *GUCA1A*^p.R120L^ and *GUCA1A*^WT+p.R120L^ (**Figure 5h**–**j**). mRNA levels of several RPE characteristic markers were reduced in *GUCA1A*^p.R120L^- and *GUCA1A*^WT+p.R120L^-injected groups when compared with the *GUCA1A*^WT^-injected group (**Figure 5k**). No statistical difference was detected between the *GUCA1A*^p.R120L^- and *GUCA1A*^WT+p.R120L^-injected groups. Thus, our findings suggest that the *GUCA1A* p.R120L mutation could cause RPE degeneration in a toxic gain-of-function manner.

### *GUCA1A* p.R120L leads to aberrant ocular vasculature

We next determined whether this mutation could cause atrophy of the choriocapillaris. Ocular vasculature in zebrafish is distinct from that in mammals. Choriocapillaris in zebrafish is not generally distinguishable. We therefore used a transgenic zebrafish (*flk1*: EGFP) model in which the expression of EGFP is driven by the promoter of the *flk1* gene, which corresponds to the *KDR* gene in humans and is expressed in vascular endothelial cells.^20,21^ This model allowed us to visualize the systemic vessels, including ocular vessels, in living zebrafish. The transgenic (*flk1*: EGFP) zebrafish were divided into two groups and injected with *GUCA1A*^WT^ or *GUCA1A*^p.R120L^. At 4 dpf, living embryos with no remarkable systemic changes were selected for fluorescent microscopy of ocular vessels. A number of consecutive fluorescent confocal images on the z axis spanning entire eyeball were automatically taken and three-dimensional reconstructions of these images were subsequently generated (**Figure 5m**). The relative fluorescent intensity of EGFP in reconstructed images, presumably derived from the retinal vessels and the choriocapillaris underneath, was calculated using ImageJ software (National Institutes of Health, Bethesda, MD). Using this approach, we found that the EGFP signal in the *GUCA1A*^p.R120L^-injected group (*n* = 5) was significantly reduced (by 71%) when compared with the *GUCA1A*^WT^-injected group (*n* = 5) (**Figure 5n**), suggesting atrophy of ocular vasculature caused by overexpression of *GUCA1A*^p.R120L^. The truncal vasculature of the living zebrafish was also obtained. Notably, no appreciable phenotypic differences were found between the truncal vasculatures of the two injected groups (**Figure 5l**), suggesting that the changes in vascular architecture are retina specific.

### *GUCA1A* p.D100E impairs photoreceptor and RPE functions in a likely dominant-negative manner

*GUCA1A* p.D100E was previously reported to be correlated with cone dystrophy; therefore, we next tried to determine whether the pathogenesis of p.D100E differed from that of p.R120L. Immunofluorescent staining revealed that staining of rhodopsin vanished in zebrafish injected with *GUCA1A*^p.D100E^ (**Supplementary Figure S2A** online), but returned in the *GUCA1A*^WT+p.D100E^-injected group (**Supplementary Figure S2B** online), which was consistent with the mRNA findings (**Supplementary Figure S2C** online). Similarly, staining of Zpr-2 was eliminated in *GUCA1A*^p.D100E^*-*injected group (**Supplementary Figure S2D** online), but could be rescued by co-injection of *GUCA1A*^WT^ (**Supplementary Figure S2E** online). In addition, mRNA expression studies revealed that the deleterious effects on RPE cells caused by p.D100E could be rescued by the WT mRNA (**Supplementary Figure S2F** online). Thus, our findings suggest that *GUCA1A* p.D100E impairs photoreceptor and RPE functions in a dominant-negative manner.

## Discussion

*GUCA1A* mutations are implicated in a wide panel of retinal dystrophies and are shown to result in constitutive activation of retGC1, which in turn leads to calcium overload, causing photoreceptor death, predominantly in cones.^26,27^ In this study we reveal a novel *GUCA1A* mutation, p.R120L, in a family with variable maculopathies ranging from mild photoreceptor degeneration to CACD. Functional analyses further support its pathogenic effects: p.R120L was shown in zebrafish to cause atrophy of photoreceptors, RPE, retinal vessels, and choriocapillaris, probably in a gain-of-function manner. Another mutation, p.D100E, previously reported to cause cone dystrophy, was also investigated. This mutation was also found to impair photoreceptors and RPE in zebrafish but, unlike p. R120L, in a dominant-negative way. In fact, diverse molecular properties of *GUCA1A* mutations have been reported. Two mutations, p.L84F and p.I107T, presenting wide phenotypic spectra, were found to cause similar abnormal regulation of retGC via disparate mechanisms.^28^ *GUCA1A* p.I107T significantly reduced the affinity for calcium binding, whereas p.L84F altered the tertiary structure of GCAP1 and aberrantly increased its affinity for magnesium. The diverse pathogenesis of *GUCA1A* mutations may contribute to their variable phenotypic spectrums.

Intrafamilial phenotypic variability has also been reported for *GUCA1A* mutations, which suggests that, apart from mutation properties, other genetic or environmental factors may also contribute to their etiology.^3^ A potential explanation is the existence of genetics modifiers. Increased expression of the mutated allele and/or decreased expression of the WT allele may aggravate retinal phenotypes. Another hypothesis is that the phenotypic severity may partly be modified by other proteins that closely interact with GCAP1 or are simply involved in the phototransduction pathway, like GCAP2, retGC1, and retGC2.^3^ In addition, a previous study also indicated that the differential photoreceptor subtype sensitivity correlated with *GUCA1A* mutations, which corresponds to its relevant distinct clinical phenotypes, may be tied to the different structural organization of rod and cone photoreceptors, the RPE, and the choriocapillaris.^29^ However, overexpression of exogenous protein in zebrafish embryos may result in unexpected phenotypes. Thus, in this study we cannot completely rule out the possibility that phenotypes observed in the RPE and ocular vessels are caused by ectopic overexpression of GCAP1. More investigations into the phenotypic diversity of *GUCA1A* mutations with a more suitable model would be worth pursuing.

CACD usually presents as an autosomal-dominant trait.^7^ Only two genes, *PRPH2* and *GUCY2D*, have been reported to cause CACD, both of which are expressed exclusively in photoreceptors, just like *GUCA1A*.^8,10,11,12^ In addition, significant thinning to complete loss of the outer nuclear layer of photoreceptors has also been observed in the CACD process.^8,30^ We therefore raise the hypothesis that CACD represents a severe pattern of maculopathy. Though the clinical recognition of CACD is mainly focused on RPE and choriocapillaris defects, these changes are probably secondary to photoreceptor dystrophies, given that all genes associated with CACD to date are photoreceptor-specific. However, no authoritative clarification has been made regarding this. In this study, by modeling *GUCA1A* p.R120L in zebrafish, we revealed that overexpression of this mutation resulted in a significant degeneration of both rods and cones, further supporting that photoreceptor dystrophy is presumably the initial defect for CACD. The greater involvement of rods observed in this study could be specific to an allele specific and/or to the species. Indeed, GCAP1 expression patterns differ among species,^31^ and GCAP1 is localized to rod cells and short single cones in zebrafish retina.^32^

However, how photoreceptor degeneration would affect the RPE and choriocapillaris is not yet clear. It is very likely that the overaccumulation of OS debris or other abnormal substances derived from those dying photoreceptors are toxic to the RPE, leading to its degeneration and atrophy. Intact RPE functions are crucial for photoreceptor survival.^33^ An impaired RPE will in turn aggravate photoreceptor degeneration. The choriocapillaris also collapses following RPE degeneration as a result of a lack of growth factors secreted by the RPE, which are essential for choroidal vasculature homeostasis. One of those crucial factors is the vascular endothelial growth factor secreted by the RPE. Targeted deletion of *Vegfa* in adult mice has been shown to induce significant retinal degeneration,^34^ further supporting its important role in maintaining homeostasis of choriocapillaries and photoreceptors. Vascular insufficiency accelerates accumulation of toxin in the Bruch’s membrane, thereby aggravating photoreceptor and RPE loss and resulting in dystrophy of the entire photoreceptor–RPE–Bruch’s membrane–choriocapillaris complex. This model implies the tight biological interplay among photoreceptors, RPE, and choriocapillaris. One example supporting our theory is that mutations in the photoreceptor-specific gene *ABCA4* impair the visual cycle and cause accumulation of abnormal toxic retinoid in the RPE, which in turn leads to Stargardt’s macular dystrophy.^35^ Similar to our cases, primary defects of photoreceptors leading to secondary RPE/choriocapillaris impairments were also revealed in mice overexpressing mutated *PRPH2* p.R172W, another photoreceptor-specific gene associated with CACD.^29^ This *PRPH2* mutation led to dominant defects in cones followed by loss of integrity of the RPE and choriocapillaris. Nevertheless, in-depth assessments of how photoreceptor degeneration impairs the RPE/choriocapillaris in variable conditions are still needed.

In summary, based on genetic findings and functional analyses, we conclude that *GUCA1A* mutations could cause significant variability of maculopathies. We also raise a theory that although clinical recognition focuses mainly on impairments of the RPE and choriocapillaris, the primary insult for CACD is in photoreceptors. CACD represents a severe pattern of maculopathy. Pathogenesis for *GUCA1A* mutations varies, which might help to explain their phenotypic diversity. Understanding the pathological process and mechanism of *GUCA1A* mutations would direct relevant clinical evaluation, treatment, and prognosis.

## Disclosure

The authors declare no conflict of interest.

## References

[bib1] Payne AM, Downes SM, Bessant DA, et al. A mutation in guanylate cyclase activator 1A (GUCA1A) in an autosomal dominant cone dystrophy pedigree mapping to a new locus on chromosome 6p21.1. Hum Mol Genet 1998;7:273–277. 9425234 10.1093/hmg/7.2.273

[bib2] Wilkie SE, Newbold RJ, Deery E, et al. Functional characterization of missense mutations at codon 838 in retinal guanylate cyclase correlates with disease severity in patients with autosomal dominant cone-rod dystrophy. Hum Mol Genet 2000;9:3065–3073. 11115851 10.1093/hmg/9.20.3065

[bib3] Michaelides M, Wilkie SE, Jenkins S, et al. Mutation in the gene GUCA1A, encoding guanylate cyclase-activating protein 1, causes cone, cone-rod, and macular dystrophy. Ophthalmology 2005;112:1442–1447. 15953638 10.1016/j.ophtha.2005.02.024

[bib4] Cuenca N, Lopez S, Howes K, Kolb H. The localization of guanylyl cyclase-activating proteins in the mammalian retina. Invest Ophthalmol Vis Sci 1998;39:1243–1250. 9620085

[bib5] Palczewski K, Subbaraya I, Gorczyca WA, et al. Molecular cloning and characterization of retinal photoreceptor guanylyl cyclase-activating protein. Neuron 1994;13:395–404. 7520254 10.1016/0896-6273(94)90355-7

[bib6] Dizhoor AM, Olshevskaya EV, Henzel WJ, et al. Cloning, sequencing, and expression of a 24-kDa Ca(2+)-binding protein activating photoreceptor guanylyl cyclase. J Biol Chem 1995;270:25200–25206. 7559656 10.1074/jbc.270.42.25200

[bib7] Boon CJ, Klevering BJ, Cremers FP, et al. Central areolar choroidal dystrophy. Ophthalmology 2009;116:771–82, 782.e1. 19243827 10.1016/j.ophtha.2008.12.019

[bib8] Smailhodzic D, Fleckenstein M, Theelen T, et al. Central areolar choroidal dystrophy (CACD) and age-related macular degeneration (AMD): differentiating characteristics in multimodal imaging. Invest Ophthalmol Vis Sci 2011;52:8908–8918. 22003107 10.1167/iovs.11-7926

[bib9] Sorsby A, Crick RP. Central areolar choroidal sclerosis. Br J Ophthalmol 1953;37:129–139. 13032369 10.1136/bjo.37.3.129 PMC1324083

[bib10] Hughes AE, Lotery AJ, Silvestri G. Fine localisation of the gene for central areolar choroidal dystrophy on chromosome 17p. J Med Genet 1998;35:770–772. 9733038 10.1136/jmg.35.9.770 PMC1051432

[bib11] Reig C, Serra A, Gean E, et al. A point mutation in the RDS-peripherin gene in a Spanish family with central areolar choroidal dystrophy. Ophthalmic Genet 1995;16:39–44. 7493155 10.3109/13816819509056911

[bib12] Hughes AE, Meng W, Lotery AJ, Bradley DT. A novel GUCY2D mutation, V933A, causes central areolar choroidal dystrophy. Invest Ophthalmol Vis Sci 2012;53:4748–4753. 22695961 10.1167/iovs.12-10061

[bib13] Zhao C, Bellur DL, Lu S, et al. Autosomal-dominant retinitis pigmentosa caused by a mutation in SNRNP200, a gene required for unwinding of U4/U6 snRNAs. Am J Hum Genet 2009;85:617–627. 19878916 10.1016/j.ajhg.2009.09.020 PMC2775825

[bib14] Zhao C, Lu S, Zhou X, Zhang X, Zhao K, Larsson C. A novel locus (RP33) for autosomal dominant retinitis pigmentosa mapping to chromosomal region 2cen-q12.1. Hum Genet 2006;119:617–623. 16612614 10.1007/s00439-006-0168-3

[bib15] Liu Y, Chen X, Xu Q, et al. SPP2 Mutations Cause Autosomal Dominant Retinitis Pigmentosa. Sci Rep 2015;5:14867. 26459573 10.1038/srep14867 PMC4602186

[bib16] Chen X, Zhao K, Sheng X, et al. Targeted sequencing of 179 genes associated with hereditary retinal dystrophies and 10 candidate genes identifies novel and known mutations in patients with various retinal diseases. Invest Ophthalmol Vis Sci 2013;54:2186–2197. 23462753 10.1167/iovs.12-10967

[bib17] Chen X, Sheng X, Liu X, et al. Targeted next-generation sequencing reveals novel USH2A mutations associated with diverse disease phenotypes: implications for clinical and molecular diagnosis. PLoS One 2014;9:e105439. 25133613 10.1371/journal.pone.0105439 PMC4136877

[bib18] Chen X, Liu Y, Sheng X, et al. PRPF4 mutations cause autosomal dominant retinitis pigmentosa. Hum Mol Genet 2014;23:2926–2939. 24419317 10.1093/hmg/ddu005

[bib19] Krock BL, Bilotta J, Perkins BD. Noncell-autonomous photoreceptor degeneration in a zebrafish model of choroideremia. Proc Natl Acad Sci USA 2007;104:4600–4605. 17360570 10.1073/pnas.0605818104 PMC1810335

[bib20] Bussmann J, Lawson N, Zon L, Schulte-Merker S; Zebrafish Nomenclature Committee. Zebrafish VEGF receptors: a guideline to nomenclature. PLoS Genet 2008;4:e1000064. 18516225 10.1371/journal.pgen.1000064 PMC2367445

[bib21] Goishi K, Klagsbrun M. Vascular endothelial growth factor and its receptors in embryonic zebrafish blood vessel development. Curr Top Dev Biol 2004;62:127–152. 15522741 10.1016/S0070-2153(04)62005-9

[bib22] Kamenarova K, Corton M, García-Sandoval B, et al. Novel GUCA1A mutations suggesting possible mechanisms of pathogenesis in cone, cone-rod, and macular dystrophy patients. Biomed Res Int 2013;2013:517570. 24024198 10.1155/2013/517570 PMC3759255

[bib23] Kitiratschky VB, Behnen P, Kellner U, et al. Mutations in the GUCA1A gene involved in hereditary cone dystrophies impair calcium-mediated regulation of guanylate cyclase. Hum Mutat 2009;30:E782–E796. 19459154 10.1002/humu.21055

[bib24] Buch PK, Mihelec M, Cottrill P, et al. Dominant cone-rod dystrophy: a mouse model generated by gene targeting of the GCAP1/Guca1a gene. PLoS One 2011;6:e18089. 21464903 10.1371/journal.pone.0018089 PMC3065489

[bib25] Makino CL, Wen XH, Olshevskaya EV, Peshenko IV, Savchenko AB, Dizhoor AM. Enzymatic relay mechanism stimulates cyclic GMP synthesis in rod photoresponse: biochemical and physiological study in guanylyl cyclase activating protein 1 knockout mice. PLoS One 2012;7:e47637. 23082185 10.1371/journal.pone.0047637 PMC3474714

[bib26] Dizhoor AM, Boikov SG, Olshevskaya EV. Constitutive activation of photoreceptor guanylate cyclase by Y99C mutant of GCAP-1. Possible role in causing human autosomal dominant cone degeneration. J Biol Chem 1998;273:17311–17314. 9651312 10.1074/jbc.273.28.17311

[bib27] Wilkie SE, Li Y, Deery EC, et al. Identification and functional consequences of a new mutation (E155G) in the gene for GCAP1 that causes autosomal dominant cone dystrophy. Am J Hum Genet 2001;69:471–480. 11484154 10.1086/323265 PMC1235478

[bib28] Marino V, Scholten A, Koch KW, Dell’Orco D. Two retinal dystrophy-associated missense mutations in GUCA1A with distinct molecular properties result in a similar aberrant regulation of the retinal guanylate cyclase. Hum Mol Genet 2015;24:6653–6666. 26358777 10.1093/hmg/ddv370

[bib29] Conley SM, Stuck MW, Burnett JL, et al. Insights into the mechanisms of macular degeneration associated with the R172W mutation in RDS. Hum Mol Genet 2014;23:3102–3114. 24463884 10.1093/hmg/ddu014 PMC4030767

[bib30] ASHTON N. Central areolar choroidal sclerosis; a histo-pathological study. Br J Ophthalmol 1953;37:140–147. 13032370 10.1136/bjo.37.3.140 PMC1324084

[bib31] Otto-Bruc A, Fariss RN, Haeseleer F, et al. Localization of guanylate cyclase-activating protein 2 in mammalian retinas. Proc Natl Acad Sci USA 1997;94:4727–4732. 9114059 10.1073/pnas.94.9.4727 PMC20792

[bib32] Imanishi Y, Li N, Sokal I, et al. Characterization of retinal guanylate cyclase-activating protein 3 (GCAP3) from zebrafish to man. Eur J Neurosci 2002;15:63–78. 11860507 10.1046/j.0953-816x.2001.01835.x PMC1363676

[bib33] Zhao C, Yasumura D, Li X, et al. mTOR-mediated dedifferentiation of the retinal pigment epithelium initiates photoreceptor degeneration in mice. J Clin Invest 2011;121:369–383. 21135502 10.1172/JCI44303 PMC3007156

[bib34] Kurihara T, Westenskow PD, Bravo S, Aguilar E, Friedlander M. Targeted deletion of Vegfa in adult mice induces vision loss. J Clin Invest 2012;122:4213–4217. 23093773 10.1172/JCI65157 PMC3484459

[bib35] Weng J, Mata NL, Azarian SM, Tzekov RT, Birch DG, Travis GH. Insights into the function of Rim protein in photoreceptors and etiology of Stargardt’s disease from the phenotype in abcr knockout mice. Cell 1999;98:13–23. 10412977 10.1016/S0092-8674(00)80602-9

