# Supplementary material for: GUCA1A mutation causes maculopathy in a five-generation family with a wide spectrum of severity
Source: Genet Med. 2017 Jan 26;19(8):945–54. doi: 10.1038/gim.2016.217 (PMC5548935; doi:10.1038/gim.2016.217)
Supplement: Supplementary Figures and Tables [file gim2016217x1.zip › SI Table S4 1020.docx]

| **Table S4. ERG responses for selected affected members representing different severities of maculopathy*.** | | | | | | |
| --- | --- | --- | --- | --- | --- | --- |
| **R/L** | **Scales** | **Normative data** | **IV:6 (Grade I)** | **III:16 (Grade II)** | **III:14 (Grade III)** | **III:18 (Grade IV)** |
| **Scotopic 3.0** | | | | | | |
| R | a [ms] | 14-22 | 18 | 22 | 19 | 20 |
|  | b [ms] | 33-46 | 39 | 46 | 42 | 45 |
|  | a-wave [μV] | 155-356 | 209 | 120 (↓) | 109 (↓) | 202 |
|  | b-wave [μV] | 290-654 | 296 | 162 (↓) | 203 (↓) | 311 |
|  | b/a | 1-3 | 1.42 | 1.35 | 1.86 | 1.54 |
| L | a [ms] | 14-22 | 18 | 21 | 20 | 20 |
|  | b [ms] | 33-46 | 39 | 45 | 42 | 45 |
|  | a-wave [μV] | 155-356 | 191 | 130 (↓) | 118 (↓) | 189 |
|  | b-wave [μV] | 290-654 | 286 (↓) | 185 (↓) | 223 (↓) | 298 |
|  | b/a | 1-3 | 1.5 | 1.43 | 1.89 | 1.58 |
| **Photopic 3.0** | | | | | | |
| R | a [ms] | 13-16 | 14 | 16 | 15 | 16 |
|  | b [ms] | 29-33 | 32 | 34 (↑) | 29 | 33 |
|  | a-wave [μV] | 26-62 | 17.1 (↓) | 11.1 (↓) | 11.7 (↓) | 11.5 (↓) |
|  | b-wave [μV] | 103-250 | 42.5 (↓) | 17.8 (↓) | 30.8 (↓) | 24.3 (↓) |
| L | a [ms] | 13-16 | 13 | 18 (↑) | 10 (↓) | 16 |
|  | b [ms] | 29-33 | 35 (↑) | 36 (↑) | 35 (↑) | 34 (↑) |
|  | a-wave [μV] | 26-62 | 9.34 (↓) | 11.3 (↓) | 13.1 (↓) | 8.51 (↓) |
|  | b-wave [μV] | 103-250 | 40.1 (↓) | 15.9 (↓) | 36.1 (↓) | 24.4 (↓) |
| **Photopic 3.0 flicker** | | | | | | |
| R | P1 [ms] | 58-64 | 68 (↑) | 70 (↑) | 66 (↑) | 68 (↑) |
|  | N1-P1 [μV] | 57-223 | 58.6 | 19 (↓) | 32.4 (↓) | 31.9 (↓) |
|  | 30 Hz Amp [μV] | - | 22.9 | 6.51 | 13.8 | 14.4 |
| L | P1 [ms] | 58-64 | 70 (↑) | 70 (↑) | 65 (↑) | 67 (↑) |
|  | N1-P1 [μV] | 57-223 | 43.5 (↓) | 23.7 (↓) | 51.6 (↓) | 34.6 (↓) |
|  | 30 Hz Amp [μV] | - | 17.3 | 8.93 | 19.9 | 14.9 |
| *Severities of maculopathy in this family were graded into stages I to IV | | | | | | |
