# Supplementary material for: GUCA1A mutation causes maculopathy in a five-generation family with a wide spectrum of severity
Source: Genet Med. 2017 Jan 26;19(8):945–54. doi: 10.1038/gim.2016.217 (PMC5548935; doi:10.1038/gim.2016.217)
Supplement: Supplementary Figures and Tables [file gim2016217x1.zip › SI Figure legends 1027.docx]

**Supplementary figure legends**

**Figure S1 ERG presentations for patients representing different severities of maculopathy.** Scotopic ERG responses are slightly reduced in patient III:16 with grade II maculopathy (**C-D**) and patient III:14 with grade III maculopathy (**E-F**).

Photopic ERG responses are obviously reduced in patient DC-IV:6 with grade I maculopathy (**A-B**), patient DC-III:16 with grade II maculopathy (**C-D**), patient DC-III:14 with grade III maculopathy (**E-F**), and patient DC-III:18 with grade IV maculopathy (**G-H**). OD = right eye; OS = left eye.

**Figure S2 Photoreceptor and RPE defects caused by *GUCA1A* p.R120L in zebrafish.** (**A-B**, **D-E**) Both RHO and Zpr-2 staining were diminished in *GUCA1A*^p.D100E^ injected zebrafish (**A**, **D**), but was re-emerged in the co-injection group (**B**, **E**). (**C**, **F**) Relative mRNA levels of photoreceptor (**C**) and RPE (**F**) characteristic transcripts in *GUCA1A*^p.D100E^ and injected groups were decreased when compared with *GUCA1A*^WT^ injected zebrafish. No statistical significance was noticed between the *GUCA1A*^WT^ and *GUCA1A*^WT+p.D100E^ injected zebrafish.
