# Supplementary figures and images for: GUCA1A mutation causes maculopathy in a five-generation family with a wide spectrum of severity
Source: Genet Med. 2017 Jan 26;19(8):945–54. doi: 10.1038/gim.2016.217 (PMC5548935; doi:10.1038/gim.2016.217)

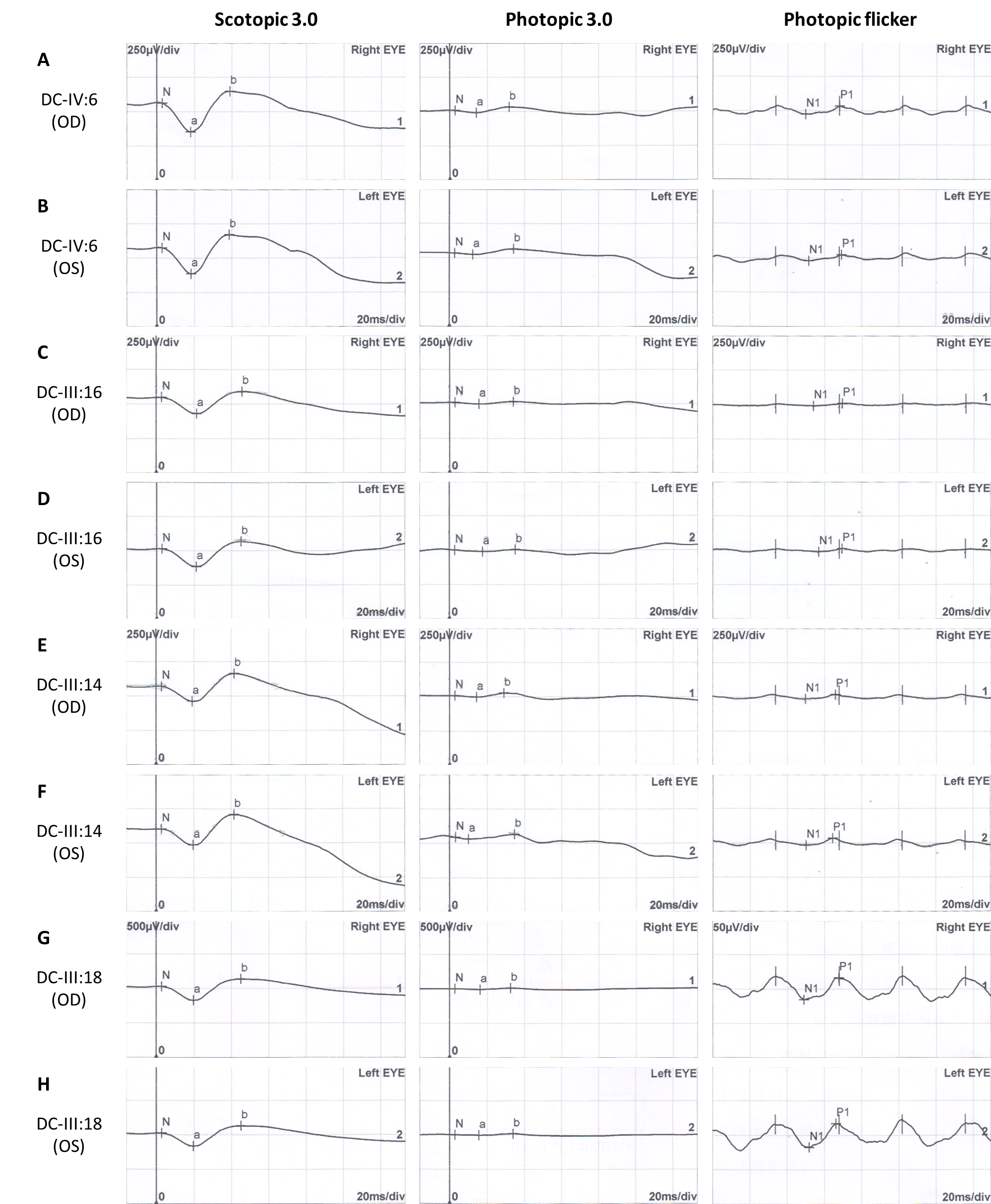

Supplement: Supplementary Figures and Tables [file gim2016217x1.zip › SI Figure S1.tif]

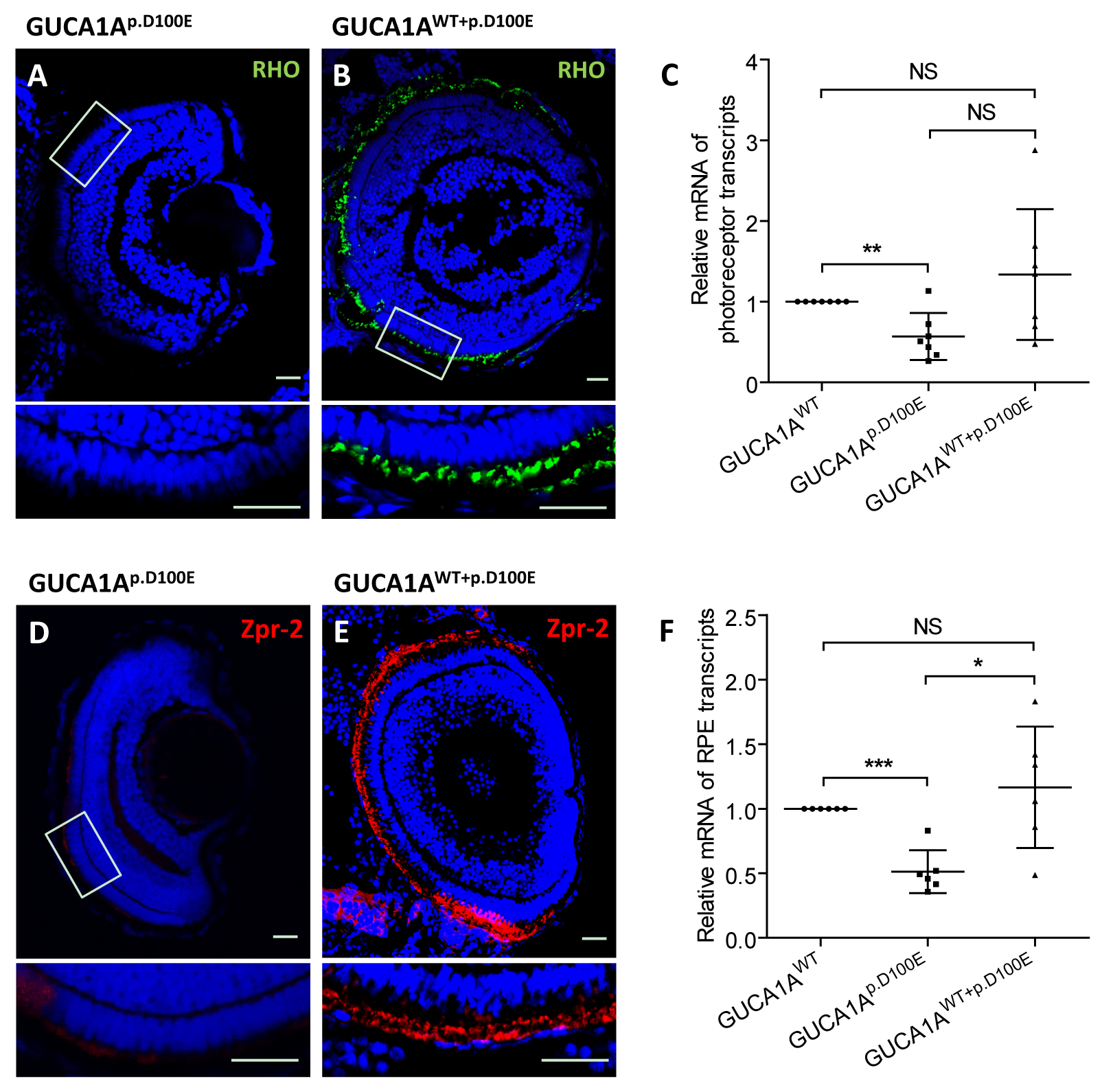

Supplement: Supplementary Figures and Tables [file gim2016217x1.zip › SI Figure S2 1027.tif]
