# Supplementary material for: GUCA1A mutation causes maculopathy in a five-generation family with a wide spectrum of severity
Source: Genet Med. 2017 Jan 26;19(8):945–54. doi: 10.1038/gim.2016.217 (PMC5548935; doi:10.1038/gim.2016.217)
Supplement: Supplementary Figures and Tables [file gim2016217x1.zip › SI Table S5 1020.doc]

| **Table S5. Details of microsatellite markers with LOD score > 0.** | | | |
| --- | --- | --- | --- |
| **Chr** | **STR Marker** | **Genetic Marshfield (cM)** | **LOD Score** |
| 3 | D3S1270 | 6.96 | 0.0257 |
| 4 | D4S2936 | 194.45 | 1.1814 |
| 4 | bt. D4S2936 & D4S2935 | 7.72 | 1.5518 |
| 4 | bt. D4S2935 & D4S403 | 19.93 | 0.6398 |
| 4 | D4S403 | 25.9 | 0.1084 |
| 4 | bt. D4S403 & D4S2994 | 30.995 | 0.2016 |
| 6 | bt. D6S276 & D6S1610 | 49.11 | 3.656 |
| 6 | D6S1610 | 53.81 | 4.5376 |
| 6 | bt. D6S1610 & D6S1549 | 57.835 | 4.4276 |
| 6 | D6S1549 | 61.86 | 4.3406 |
| 6 | bt. D6S1549 & D6S452 | 67.495 | 3.9521 |
| 6 | D6S452 | 73.13 | 3.539 |
| 6 | bt. D6S452 & D6S460 | 81.48 | 2.396 |
| **Abbreviations:** Chr: chromosome; LOD: log odds score; STR: short tandem repeats; cM: centi Morgan; bt.: between. | | | |
