# Supplementary material for: GUCA1A mutation causes maculopathy in a five-generation family with a wide spectrum of severity
Source: Genet Med. 2017 Jan 26;19(8):945–54. doi: 10.1038/gim.2016.217 (PMC5548935; doi:10.1038/gim.2016.217)
Supplement: Supplementary Figures and Tables [file gim2016217x1.zip › SI Table S1 0701.doc]

| **Table S1. Summary of Primer Information** | | |
| --- | --- | --- |
| **Gene** | **Forward Primer (5'→3')** | **Reverse Primer (5'→3')** |
| ***For Exome Sequencing Analysis*** | | |
| *GUCA1A* | CTCTTGAGTCCCAGCTCTGC | CCACGTGAGGAGAAGTCAGA |
| *MYOD1* | ACGGCTCTCTCTGCTCCTTT | GTTTGGATTGCTCGACGTG |
| *ELP4* | GAGACCCTCAACCAGGACAC | CGCTCTGAACCGACCTAAGA |
| *CDC27* | AGTGCAGCCCATAATTCACA | CCACGAGAGTCACTATCTGCCTA |
| *FLNB* | GCGCATCTGACAGTTGAAGA | CACACCAATGACCAGGACAC |
| *CREB3L2* | AGCTCTCCTGCATGTGTCTTC | AAAGCCAGCTACAACATCTGTG |
| *CASP2* | GGTTAAATGCCTTCTGTGAGC | TGGTCCCAAAGTGTTGTTCA |
| *SSPO* | GAGTGAGTGGTCCGAGTGGT | GGCCTTAGAGACAAGCAGGA |
| *TMEM176A* | CCCTGAGTCCATCCTTTCCT | GAACAGCACTGGGAAGGGTA |
| *ABCA1* | TCAGCTATGCCTGCTGCTATT | AACCCAGACAGTGGCATGA |
| ***For Expression Study in Zebrafish*** | | |
| *β-actin* | CGAGCTGTCTTCCCATCCA | TCACCAACGTAGCTGTCTTTCTG |
| *rho* | CGTGCACTTCTTCATCCCAC | AGGAAGGCGATGACCATGAT |
| *prph2b* | ACCACCTGACCAACAACTCC | CAACCCTCACTTTCGCTCTC |
| *rs1* | CCTGAAGGAGGTGAAAGTGG | TGGAAGAGCGGTCAGAGTTC |
| *opn1sw1* | TTCCTTCTCGTCACCTGCTT | AATCCTTGTTTGGCTCATCG |
| *arr3l* | GGGATCCAATCCCTGTCAAT | GCCACGTTTCTCCTTGTTGT |
| *gnat2* | AGTCTGCTCTGGCCATCATC | AGCAGAGTCGTTCAGCTGGT |
| *opn1lw1* | AAACCACAAGGGAAGCAATG | GGATCCAGTTGAGAGGGTGA |
| *rpe65c* | ATCCCGCCACTACAAGAAGA | TGCCAAAGCTGTGAACGTAG |
| *rlbp1b* | TGGACATGTTGCAGGACTCT | ACAAACACCCTTTCCAGCAG |
| *lrat* | TCAGACCTACCAGTTCTGCAA | CAAACAGGGTCACCGACTG |
| *mertk* | GCTACATCGCGGTCAGAGAG | AACGCCCTCAGTGGTGTTAC |
| *best1* | GTGGGCTGGCTTAAGGTTG | CTGACAGCAGAGACACCTGTAAA |
| *ctnnb1* | GGGATCAAATCTGACACCAAA | CCTCAGCTCCCTGGTCAAT |
