# Supplementary material for: GUCA1A mutation causes maculopathy in a five-generation family with a wide spectrum of severity
Source: Genet Med. 2017 Jan 26;19(8):945–54. doi: 10.1038/gim.2016.217 (PMC5548935; doi:10.1038/gim.2016.217)
Supplement: Supplementary Figures and Tables [file gim2016217x1.zip › SI Table S2 0921.doc]

| **Table S2. Antibodies Used in This Study** | | | |
| --- | --- | --- | --- |
| **Antibodies** | **Host** | **Dilution (Application)** | **Supplier (catalog number)** |
| Rhodopsin | Mouse | 1:250 (Immunofluorescence) | Abcam (ab5417) |
| PNA | / | 1:200 (Immunofluorescence) | Invitrogen (PN40265) |
| Zpr-1 | Mouse | 1:250 (Immunofluorescence) | ZRIC (ZDB-ATB-081002-43) |
| Zpr-2 | Mouse | 1:250 (Immunofluorescence) | ZRIC (ZDB-ATB-081002-44) |
