# Supplementary material for: GUCA1A mutation causes maculopathy in a five-generation family with a wide spectrum of severity
Source: Genet Med. 2017 Jan 26;19(8):945–54. doi: 10.1038/gim.2016.217 (PMC5548935; doi:10.1038/gim.2016.217)
Supplement: Supplementary Figures and Tables [file gim2016217x1.zip › SI Table S3 1027.doc]

| **Table S3. Clinical features of included patients** | | | | | | | | | | |
| --- | --- | --- | --- | --- | --- | --- | --- | --- | --- | --- |
| **Patient ID** | **Sex** | **Age**  **(year)^*^** | **Onset Age**  **(year)^†^** | **CA** | **BCVA (logMAR)** | | **Refractive Errors** | | **Grades of**  **Maculopathy** | |
|  |  |  |  |  | **O.D.** | **O.S.** | **O.D.** | **O.S.** | **O.D.** | **O.S.** |
| DC-II:6 | F | 70 | Early 20s | Yes | FC | FC | +2.25DS/+0.75DC*15 | +1.25DS/+1.20DC*75 | IV | IV |
| DC-II:11 | F | 66 | Early 30s | Yes | 0.12 | 0.1 | +0.25DS/+0.50DC*85 | +1.75DS/-2.00DC*165 | III | IV |
| DC-II:13 | F | 60 | Late 20s | Yes | FC | FC | +0.75DS/-0.75DC*40 | +0.25DS/+1.00DC*5 | III | III |
| DC-III:7 | M | 60 | 27 | Yes | FC | FC | +0.75DS/-1.75DC*100 | +0.25DS/+0.75DC*175 | IV | IV |
| DC-III:12 | M | 49 | 33 | Yes | 0.12 | 0.1 | +1.00DS/-0.50DC*5 | +0.50DS/+0.75DC*125 | III | III |
| DC-III:14 | M | 57 | 40 | Yes | 0.1 | FC | +1.50DS/-1.50DC*85 | +0.25DS/+0.75DC*180 | III | III |
| DC-III:16 | F | 55 | 30 | Yes | 0.4 | 0.5 | +1.25DS/-1.75DC*15 | +0.25DS/-1.00DC*95 | II | II |
| DC-III:18 | M | 53 | 24 | Yes | 0.1 | 0.08 | +0.25DS/-0.50DC*85 | +0.25DS/-0.50DC*85 | IV | IV |
| DC-III:20 | F | 50 | 39 | Yes | 0.5 | 0.4 | +0.50DS/-1.00DC*20 | +1.50DS/-1.00DC*90 | II | II |
| DC-III:31 | M | 43 | 38 | Yes | 0.6 | 0.8 | -1.00DS | -1.00DS/-0.50DC*125 | I | I |
| DC-III:33 | M | 41 | 28 | Yes | 0.12 | 0.2 | +1.25DS/-0.50DC*25 | +0.25DS/-1.50DC*95 | III | III |
| DC-IV:3 | F | 35 | 29 | Yes | 0.12 | 0.12 | -0.25DS | -0.50DC*50 | III | III |
| DC-IV:5 | F | 35 | 32 | Yes | 0.12 | 0.15 | +0.25DS/-0.25DC*110 | -0.50DC*80 | III | III |
| DC-IV:6 | M | 31 | 29 | Yes | 0.6 | 1.0 | -0.75DS/-0.50DC*110 | -1.25DS/-0.50DC*130 | I | I |
| DC-IV:9 | F | 33 | - | No | 1.0 | 1.0 | -0.75DS/-0.25DC*95 | -0.25DS/-0.50DC*105 | I | I |
| DC-IV:13 | F | 31 | 23 | Yes | 0.5 | 0.25 | -1.75DS/-0.50DC*120 | -2.50DS/-0.5DC*180 | III | III |
| DC-IV:19 | M | 18 | ? | Yes | 0.6 | 0.6 | -2.50DS/-0.25DC*175 | -1.75DS/-0.50DC*90 | II | II |
| DC-V:3 | M | 13 | - | No | 1.0 | 1.0 | -0.50DS/-0.25DC*130 | -1.25DS | I | I |
| *: Age of recruitment and examination; †: Onset age of vision loss; -: Patient has not reported vision loss yet; ?: This patient reported to have poor vision since early childhood. Therefore, it is difficult to determine his onset age for vision loss.  **Abbreviations:** CA: color anomaly; BCVA: best corrected visual acuity; O.D.: right eye; O.S.: left eye; FC: finger count. | | | | | | | | | | |
